# Integral analysis of p53 and its value as prognostic factor in sporadic colon cancer

**DOI:** 10.1186/1471-2407-13-277

**Published:** 2013-06-05

**Authors:** Arantza Fariña Sarasqueta, Giusi Forte, Wim E Corver, Noel F de Miranda, Dina Ruano, Ronald van Eijk, Jan Oosting, Rob AEM Tollenaar, Tom van Wezel, Hans Morreau

**Affiliations:** 1 Department of Pathology, Leiden University Medical Centre, P.O. Box 9600 L1-Q2300 RC, Leiden, the Netherlands; 2 Department of Surgery, Leiden University Medical Centre, Leiden, the Netherlands

**Keywords:** Colon cancer, p53, Prognosis, Survival, CSKN1A1

## Abstract

**Background:**

p53 (encoded by *TP53*) is involved in DNA damage repair, cell cycle regulation, apoptosis, aging and cellular senescence. *TP53* is mutated in around 50% of human cancers. Nevertheless, the consequences of p53 inactivation in colon cancer outcome remain unclear. Recently, a new role of p53 together with CSNK1A1 in colon cancer invasiveness has been described in mice.

**Methods:**

By combining data on different levels of p53 inactivation, we aimed to predict p53 functionality and to determine its effects on colon cancer outcome. Moreover, survival effects of *CSNK1A1* together with p53 were also studied.

Eighty-three formalin fixed paraffin embedded colon tumors were enriched for tumor cells using flow sorting, the extracted DNA was used in a custom SNP array to determine chr17p13-11 allelic state; p53 immunostaining, *TP53* exons 5, 6, 7 and 8 mutations were determined in combination with mRNA expression analysis on frozen tissue.

**Results:**

Patients with a predicted functional p53 had a better prognosis than patients with non functional p53 (Log Rank p=0.009). Expression of *CSNK1A1* modified p53 survival effects. Patients with low *CSNK1A1* expression and non-functional p53 had a very poor survival both in the univariate (Log Rank p<0.001) and in the multivariate survival analysis (HR=4.74 95% CI 1.45 – 15.3 p=0.009).

**Conclusion:**

The combination of mutational, genomic, protein and downstream transcriptional activity data predicted p53 functionality which is shown to have a prognostic effect on colon cancer patients. This effect was specifically modified by *CSKN1A1* expression.

## Background

During colon carcinogenesis cells accumulate several genetic and genomic aberrations that lead to uncontrolled proliferation and tumor formation [1]. A major event in the adenoma to carcinoma transition is *TP53* inactivation. p53 plays a crucial role in maintaining genome stability and integrity. Upon DNA damage, the activation of p53 leads to cell cycle arrest enabling the cells to repair the damaged DNA. On the other hand, when the damage is too extensive to be repaired p53 activation can also drive the cell towards apoptosis or senescence [2]. Recently, p53 has also been implicated in tumor invasiveness [3]. In mice, the inactivation of casein kinase 1 alpha (*Csnk1a1*) promotes the cytoplasmatic/nuclear accumulation of β catenin which stimulates the transcription of *Wnt* signaling target genes. The combined inactivation of *p53* and *Csnk1a1* rapidly leads to tumor invasiveness in the colon of these mice.

Inactivation of *TP53* is one of the most frequent events in human cancer [4]. Among others, *TP53* can be inactivated by “loss of function” mutations in one allele and deletion of the remaining wild type allele or by dominant negative mutations that are able to inactivate also the wild type protein transcribed by the second unaffected allele. Either way, when p53 function is jeopardized, genomic instability and uncontrolled cell proliferation are facilitated.

The role of p53 inactivation in colon cancer progression and prognosis has been widely studied but remains elusive notwithstanding the amount of reports addressing this subject [5-17]. Chromosomal instability (CIN) is a known prognostic factor in colon cancer [18]. Although p53 inactivation has been frequently associated with CIN, not all tumors with CIN carry an inactive p53 and vice versa [19]. More complexity is added by the recent demonstration that *TP53* can behave as a haploinsufficient tumor suppressor gene. Using mouse models, Ventakachalam and coworkers demonstrated that mice carrying one functional *p53* allele developed tumors but they showed however a milder phenotype than mice that lost both alleles [20]. Moreover, several reports described the *TP53* gene dosage effect on expression of target genes [21,22].

Recent developments in genomic copy number analysis have shown to more accurately study the measure of chromosomal structural and numeric aberrations [23]. The development of the lesser allele intensity ratio (LAIR) algorithm that integrates the DNA index in the analysis of copy number data gives a real measure of the chromosomal alterations and allows the study of gene dosage effects in tumors.

Given the complexity of the p53 network, the several ways of p53 inactivation, and the recently described role of p53 in cancer invasiveness in mice, we studied in detail different levels of p53 inactivation in human colon cancer taking into account the allelic state of the locus on the short arm of chromosome 17, gene mutation state, protein expression levels, downstream target gene expression and determine the prognostic impact in colon cancer patients. Moreover, interactions with the recently described *CSNK1A1* expression and the impact on disease outcome were also explored.

## Patients and methods

### Patients

Inclusion criteria for this study were sporadic colorectal cancers in stage I, II and III. Stage IV patients were not included because the disease is metastasized and therefore the therapy has a palliative character instead of a curative character.

Thus, eighty-three sporadic colorectal cancer patients diagnosed as stage I, II or III at the Leiden University Medical Centre between 1991 and 2005 were selected for the present study. Microsatellite instability of these cancers had been determined for this group, as described elsewhere [24]. The use of clinical material was approved by the medical ethical board of the Leiden University Medical Centre

Tumors were classified according to the WHO classification of tumors of the digestive system [25].

## Methods

### Determination of p53 functionality

#### Tissue preparation for multiparameter flow cytometry and sorting

Tumor and stromal cells were sorted from FFPE tissue blocks using the FACS ARIA I (BD Biosciences, San Jose, CA, USA) based on vimentin, keratin expression and DNA content as previously described by Corver *et al.*[26,27]. DNA index (DI) defined as the ratio between the median G_0_/G_1_ keratin positive epithelial fraction and the median G_O_/G_1_ vimentin stromal fraction, was calculated using a remote link between Winlist and ModFit (Verity Software House) for each sample. Whenever, more than one keratin positive population was seen, it was independently sorted. DI was categorized as DI< 0.95; DI=0.95 – 1.05; DI=1.06 – 1.4; DI=1.41 – 1.95 and DI>1.95.

DNA was purified from sorted cells after an overnight proteinase K digestion using the Nucleospin Tissue kit (Macherey Nagel, Düren, Germany) following manufacturer’s instructions.

#### SNP array hybridization for allelic state determination

A custom Golden Gate genotyping panel with 384 SNPs was designed using the Assay Design Tool (Illumina Inc. San Diego, CA, USA). The panel contains SNPs mapping to the following chromosomes: 1q21-25, 8q22-24, 13q12-34, 17p13-11 (the *TP53* locus), 18q12-22, 20q11-13, all of which are associated with tumor progression in the colorectum [28]. SNPs on chromosome 2 served as controls. Paired samples were analysed in the Golden Gate assay as described [29] and hybridized to Sentrix Array Matrix with 384 bead types. SNP arrays were analysed in the BeadarraySNP package. The data generated was analyzed with the LAIR algorithm [23] that integrates the DNA index into the analysis. Four observers determined LAIR scores independently (AFS, WEC, GIF and TVW). FISH validated the 3 of the 83 samples that showed discordance (3.6%) between the observers.

**Figure 1:**
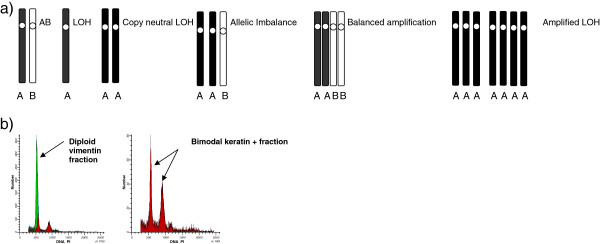
**a) Schematic representation of the possible allelic states according to LAIR scores b) Example of a DNA histogram of one tumor containing two populations with different DNA indexes.** Green histogram is the DNA diploid vimentin positive stromal fraction and in red the keratin positive epithelial fraction.

We differentiated the following allelic states:

1) Retention with genotype AB; 2) Loss of heterozygosity (LOH), genotype A; 3) copy neutral LOH (cnLOH), genotype AA; 4) amplified LOH (aLOH) genotypes AAA or AAAA etc.; 5) allelic imbalance (AI) or genotypes AAB, AAABB etc.; 6) balanced amplification (BA), genotypes AABB, AAABBB etc.; 7) multiclonal tumors (identified through flow cytometry, see Figure 1a and b) [23].

#### FISH

To confirm the copy number results obtained with the SNP array, FISH in nuclei obtained from FFPE material of seven patients was performed. First, 2mm. punches (Beecher Instruments, Silver Springs, MD, USA) of selected tumor areas were embedded in blanco acceptor paraffin blocks. Subsequently, 50 μM slices were obtained, deparaffinized and rehydrated. Antigen retrieval was performed by high pressure cooking in Tris-EDTA pH=9. After incubation for one hour at 37°C with RNAse, samples were digested with 0.5% pepsin pH=2 at 37°C for 30 minutes. The obtained nuclei were then washed and resuspended in methanol: acetic acid in a 3 to 1 proportion. Thereafter nuclei were spun onto clean glasses and hybridization with Vysis® TP53/CEP17 FISH probe kit (Abbot Molecular, IL, USA) was allowed overnight at 37°C. After washing, samples were mounted with Vectashield® mounting medium containing DAPI (Vector Laboratories Inc., Burlingame, CA, USA) and nuclei were evaluated under the fluorescence microscope.

Seven tumors were tested for which enough material was available and with different allelic states of chr.17p according to the SNP array analysis.

#### p53 IHC staining

Tissue microarrays (TMA) of these tumors were prepared by punching three representative tumor areas selected by a pathologist (HM) on HE stained slides and arraying them on a recipient paraffin block (Beecher Instruments, Silver Springs, MD, USA). Five μM slices were then cut. Heat induced antigen retrieval (HIAR) was performed as described elsewhere [28] and staining was carried out with the mouse anti-human monoclonal antibodies directed against p53 (clone D0-7, 1:1000 dilution) (Lab Vision NeoMarkers, Fremont, CA, USA).

p53 was scored in four different categories based on any level of nuclear staining, like previously described [30] by an experienced pathologist (HM) and a pathology resident (AFS): completely negative; 1- 25% positive nuclei (indicative of a wild type state); 25-75% positive nuclei and >75% positive nuclei. For analysis purposes, the last two categories were fused in only one category; more than 25% positive cells (indicative of a mutated gene).

#### TP53 mutation analysis

Tumor DNA available from 40 patients was isolated from enriched tumor areas containing at least 50% tumor cells, as described above. Four different PCRs were performed for amplification of exons 5, 6, 7 and 8 of the *TP53* gene. Ten nanograms DNA was used for each PCR using primers already published modified for SYBRgreen® detection [31]. Subsequently, PCR products were purified using Qiagen’s MinElute™96 UF PCR Purification Kit (Qiagen Sciences, Germantown, MD, USA) and reactions were sequenced using the MI13 forward and reverse primers. Analysis was performed using the Mutation Surveyor 3.97® sequence analysis and assembly software (SoftGenetics LLC, Stage College, PA, USA).

#### mRNA expression arrays

Fresh frozen tissue of fifty-seven patients was available for mRNA expression analysis. mRNA was isolated, labeled and hybridized to customized Agendia 44 K oligonucleotide array as described elsewhere [24].

### Statistical analysis

Associations between categorical variables were studied by χ^2^ and Fischer exact test. Univariate survival analysis was performed by Kaplan Meier analysis and differences between survival curves were studied by Log Rank analysis. Cox Proportional Hazard Model performed multivariate survival analysis. Cancer Specific Survival was defined as the time between curative intended surgery and death by cancer related causes [32]. Results were considered significant when p value <0.05. All tested were two tailed. All of the analyses mentioned above were performed using SPSSv16 package for Windows (Chicago, IL, USA)

Statistical analysis of the mRNA expression data was done using the LIMMA (Linear Modelling for Microarray Analysis) framework in Bioconductor [33].

The expression of the 35 genes reported by Yoon *et al.*[22] as genes which expression is *TP53* gene dosage dependent was analyzed in relation with p53 functional state. Furthermore, expression levels of three probes targeting different locations in the 3’UTR of the *CSNK1A1* gene (NM_001025105.1 transcript) were independently analyzed.

Finally, expression levels of eight genes reported by Elyada *et al.*[3] as involved in murine tumor invasiveness were also analyzed.

## Results

### Patients’ description

Patients’ characteristics are shown in Table 1. Summarized, 54% of the patients were female, 63% of the tumors were right sided (i.e. tumors located in the colon from the cecum until the splenic flexure) and 37% left sided. 4% of the patients had stage I disease at diagnosis, 61% stage II and 35% stage III. Twenty-seven tumors were MSI-H (33%), whereas 55 (67%) were MSS tumors.

**Table 1:** Patients’ characteristics

**Characteristics**	**Total N (%)**
**Age**	
50-59	14 (17)
60-69	27 (33)
70-79	24 (30)
80-89	16 (20)
**Gender**	
Male	34 (41)
Female	45 (54)
**Tumor Location**	
Right	52 (63)
Left	31 (37)
**Stage**	
I and II	54 (65)
III	29 (35)
**MMR status**	
MSS	55 (67)
MSI-H	27 (33)
**Chr.17p allelic state**	
AB	39 (47)
LOH	9 (11)
CNLOH	11 (13)
ALOH	7 (8)
AI	3 (4)
Multiple clones	14 (17)
**DNA index**	
0.95 – 1.05	35 (46)
1.06 – 1.40	10 (13)
1.41 – 1.95	31 (41)
***TP53***	
wt	22 (55)
mut	18 (45)
**IHC p53**	
0 %	10 (13)
>0% - ≤25%	35 (46)
>25%	31 (41)
**Median Follow up in months (range)**	68.84 (2–199)

Median follow up was 69 months (range 2 – 199). At the end of the follow up, 41% of the patients were alive, 24% of the patients had died because of cancer related causes and 30% died because of non-cancer related causes.

### Allelic state

**Figure 2:**
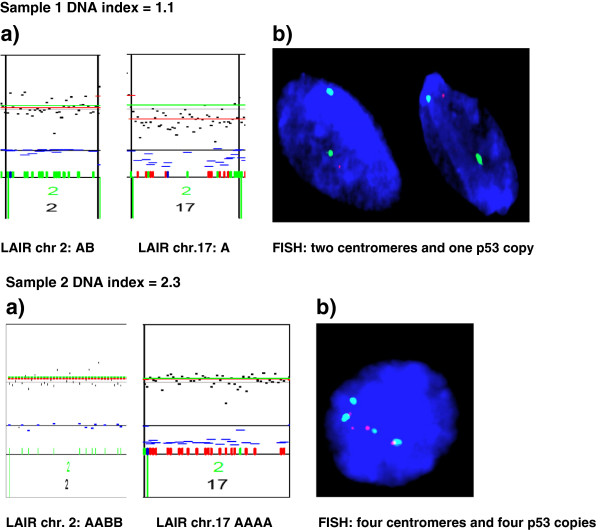
Results of a) SNP array on reference chromosome and chr.17p b) FISH on Chr. 17 (the green signal corresponds to the centromere probe and the red signal to the p53 probe).

All samples were flow cell sorted as previously described and analyzed with a custom SNP array comprising several chromosomal regions previously reported to be implicated in colorectal cancer progression [28]. In the present study we have focused on the allelic state of the *TP53* locus on chromosome 17p13-11 Of the 83 tumors analyzed, 47% were classified as normal with genotype AB, 11% as LOH (genotype A), 13% as cnLOH (genotype AA), 8% as aLOH (genotype AAA/AAAA) and 4% as AI (genotype AAB/AAABB). Note also that 17% of the patients showed multiple cancer populations by flow cytometry (results shown in Table 1). No balanced amplifications were seen in the monoclonal series. FISH analysis was used to confirm the chromosome 17 LAIR scores for seven samples (Figure 2).

### Predicted p53 *functionality*

We predicted the functionality of p53 (hereafter called functionality) for each sample (see Additional file 1: Table S1) by combining data from the *TP53* locus allelic state, mutation data and protein expression levels. Overall, the three parameters were mostly in agreement with each other, except for 6 out of 57 patients where there was one discordance between mutation state, protein expression and/or allelic state. To call p53 non functional, at least two parameters should point in that direction. Mutation state or IHC expression level weighted more in decision making whenever one of the three parameters was not available. Associations between p53 functionality and the different variables are shown in Table 2. In summary, the majority of tumors with a functional p53 (78%) lacked *TP53* mutations (p=0.01) and all showed between 0-25% positive stained cells using immunohistochemistry (p<0.001). 78% of the tumors with functional p53 had a near diploid DNA index raging from 0.95-1.05 whereas 63% of the non functional p53 samples was highly aneuploid with DNA indexes ranging 1.41 – 1.95 (p<0.001). Samples with functional p53 showed significantly more retention of the p53 locus (genotype AB) as compared to the group with either aLOH (AAA/AAAA) (p=0.005), cnLOH (AA) (p<0.001) and cases with multiclonallity (p=0.006). Moreover, the frequency of functional p53 was increased in tumors with LOH than with cnLOH (p=0.01). Furthermore, tumors with a functional p53 were significantly overrepresented in the group of right-sided tumors (p=0.035). Of the tumors with non-functional p53, eighty-six percent showed the MSS phenotype (p=0.009).

**Table 2:** Associations between clinicopathological variables and p53 functionality

	***p53 non functional N (%)***	***p53 functional N (%)***	**p value**
***TP53 mutational status***			
wt	7 (33)	14 (78)	**0.01**
mut	14 (67)	4 (22)	
**P53 IHC**			
0	3 (11)	7 (24)	**<0.001#**
0 - ≤25%	1 (3)	22 (76)	
>25%	24 (86)	0 (0)	
**Chr. 17 p status**			
AB	5 (18)	22 (76)	<0.001*
LOH	2 (7)	4 (14)	
Copy neutral LOH	9 (32)	0 (0)	
Amplified LOH	5 (18)	1 (3)	
Allelic Imbalance	1(4)	0 (0)	
Two clones	6 (21)	2 (7)	
**Age category**			
50 – 59	4 (14)	6 (22)	NS
60 – 69	10 (36)	9 (32)	
70 – 79	10 (36)	9 (32)	
80 – 89	4 (14)	4 (14)	
**DNA index**			
0.95 – 1.05	6 (22)	21 (78)	**<0.001¶**
1.06 – 1.4	4 (15)	3 (11)	
1.41 – 1.95	17 (63)	3 (11)	
**MMR status**			
MSI	4 (14)	14 (50)	**0.009**
MSS	24 (86)	14 (50)	
**Gender**			
Male	12 (43)	18 (62)	NS
Female	16 (57)	11 (38)	
**Tumor Location**			
Right	10 (36)	19 (66)	**0.035**
Left	18 (64)	10 (34)	
**Stage**			
I and II	14 (50)	22 (76)	0.06
III	14 (50)	7 (24)	
**Median Follow up in months**	66.75	89.77	**0.4**

*Χ^2^ test allelic status AB vs. LOH p=0.58; **AB vs. CNLOH p<0.001**; **AB vs. ALOH p=0.005**; **AB vs. two clones p=0.006**

**LOH vs. CNLOH p=0.01**; LOH vs. ALOH p=0.24; LOH vs. two clones p=0.28.

ALOH vs. CNLOH p=0.43; Amp LOH vs. two clones p=1.

CNLOH vs. two clones p=0.48.

# Χ^2^ test p53 IHC 0 vs. 0-25% p=0.07; **0 vs. >25% p<0.001; 0-25% vs. >25% p=0.001.**

¶ Χ^2^ test DNA index 0.95 – 1.05 vs. 1.06 – 1.4 p=0.16; **0.95 – 1.05 vs. 1.41- 1.95 p<0.001;** 1.06 – 1.40 vs. 1.41 – 1.95 p=0.29.

To corroborate the classification in functional and non-functional p53 groups, we compared p53 target gene expression levels between these two groups. We selected genes for which expression was previously shown to be p53 gene dosage dependent by Yoon *et al.*[22]. Eight genes differently expressed between both groups were identified (Table 3). As expected, known p53 targets like *MDM2* and *CDKN1A* were significantly higher expressed in the p53 functional group than in the non functional group (p=0.0025 and p=0.0013 respectively). Genes higher expressed in the non functional group were involved in many processes such as cell proliferation (*PRKCZ*), protein ubiquitination (*SIAH1*), metabolism (*HMGCS1*) and cell differentiation (*PRKCZ, PDE6A*).

**Table 3:** List of genes differentially expressed between functional p53 and non functional p53 groups

***Gene name***	***Chr. position***	***Gene description***	**p-value*, *p53 functional *vs., *p53 non functional**
***PRKCZ***	1p36.33-p36.2	Serine threonine kinase involved in several processes such as proliferation, differentiation and secretion.	4.95E-04
			↑non functional
***LMO3***	12p12.3	Lim domain only 3 (rhombotin like 2). Expression of LMO-3 represses p53 mediated mRNA expression of target genes.	1.2E-02
			↑non functional
***CDKN1A***	6p21.2	Cyclin dependent kinase inhibitor. Causes cell cycle arrest in the presence of DNA damage.	1.3E-02↑functional
***PDE6A***	5q31.2-q34	Phosphodiesterase 6A, cGMP-specific, rod, alpha	7.47E-02
			↑non functional
***SIAH1***	16q12	Seven in absentia homolog 1. Involved in ubiquitination and proteosome related degradation of specific proteins like beta catenin.	2.60E-02
			↑non functional
***TPD52L2***	20q13.2-q13.3	Tumor protein D52 like 2. Expressed in childhood leukemia and testes.	4.65E-02
			↑non functional
***MDM2***	12q14.3-q15	MDM2 p53 binding protein homolog (Mouse)	1.25E-02
			↑ functional
***HMGCS1***	5p14-p13	3-hydroxy 3-methylglutaryl-CoA synthase I	1E-01
			↑functional

All p-values are corrected for multiple tests.

### Survival analysis

**Figure 3:**
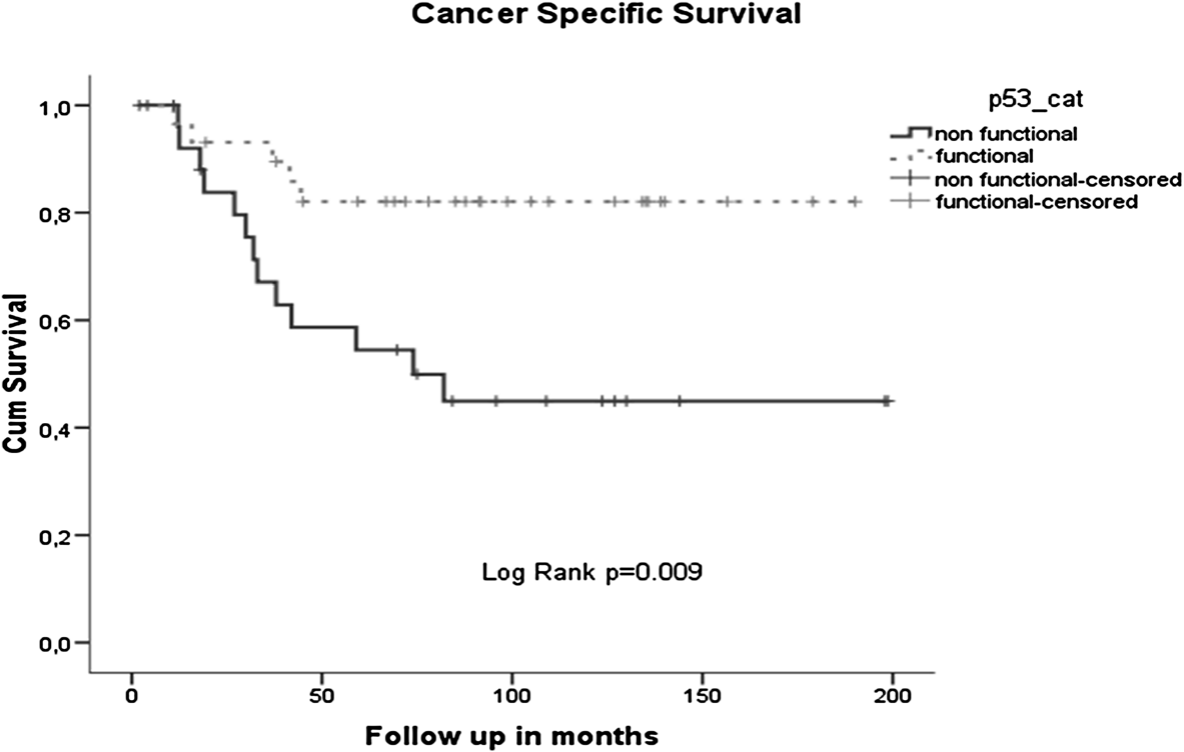
Kaplan Meier plots for CSS according to p53 functionality.

In a univariate survival analysis, p53 functionality was prognostic; patients with functional p53 had a better cancer specific survival than patients with non-functional p53 (Log rank p=0.009) (Figure 3).

In our cohort, patients with MSI-H tumors are slightly more frequent than expected from epidemiological studies (33% *vs.* 18% expected), nevertheless MMR status did not influence survival (data not shown) nor the effects of p53 functionality on survival.

Recently, the role of *p53* and *Csnk1a1* inactivation in tumor invasiveness in mice has been demonstrated [3]. We analyzed whether the expression levels of *CSNK1A1* modulate p53 effects in disease outcome. Patients were divided according to the expression level. In the group with high *CSNK1A1* expression the expression level of the three probes analyzed (A_23_P213551; A_24_P183292; A_24_P251899) exceeded the median value for that specific probe while in cases with low *CNSK1A1* expression the value was lower than the median.

**Figure 4:**
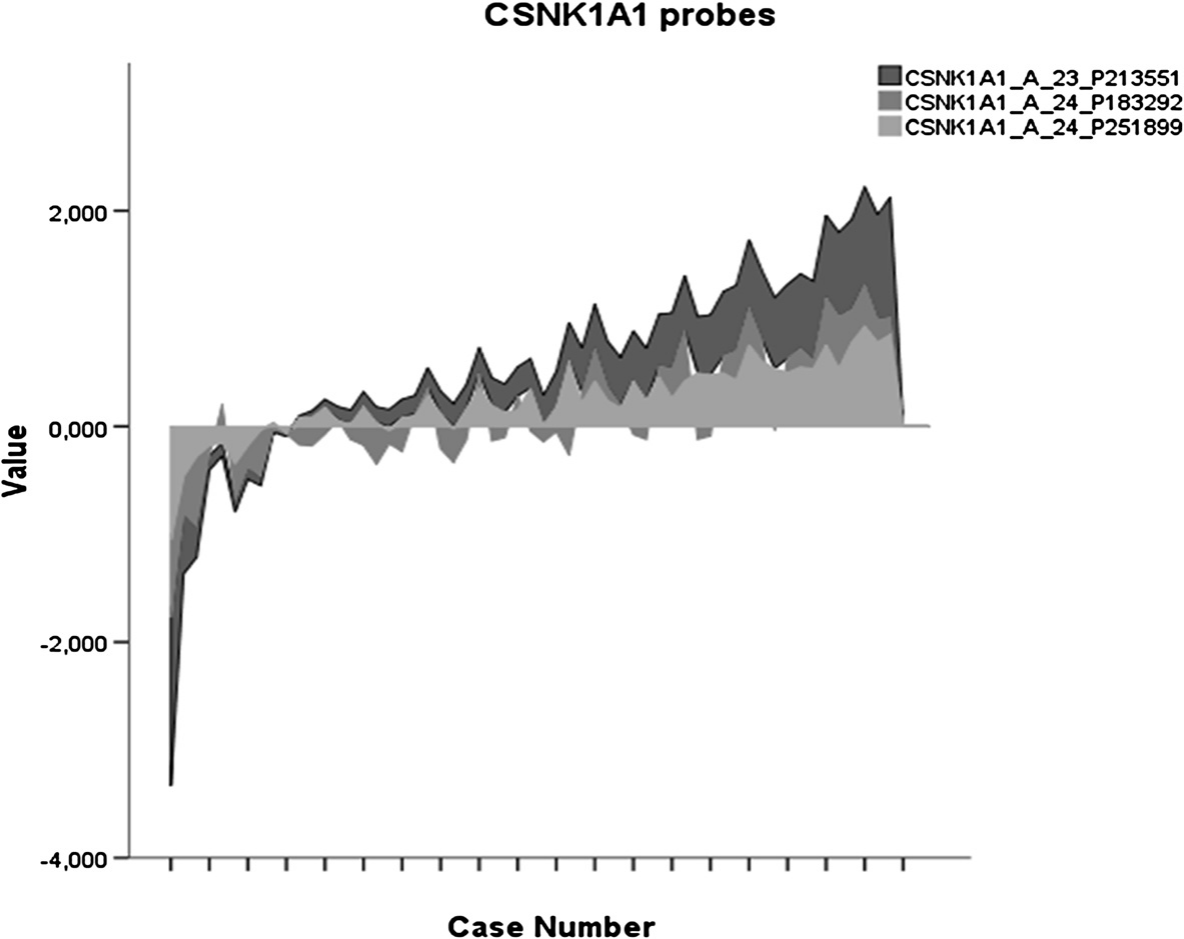
**Trends in expression of the three *CSNK1A1 *probes.**

The values of the three probes correlated significantly with each other (Pearson’s correlation coefficient =0.94 p<0.001 between A_23_P213551 and A_24_P251899, 0.747 p<0.001 between A_23_P213551 and A_24_P183292 and finally 0.743 p<0.001 between A_24_P183292 and A_24_P251899) (Figure 4). The three probes had the same detrimental effect on survival in a univariate analysis with different significant p values (data not shown). We selected the probe (A_24_P183292) with the most significant results (Log rank p=0.003) for further analyses.

**Figure 5:**
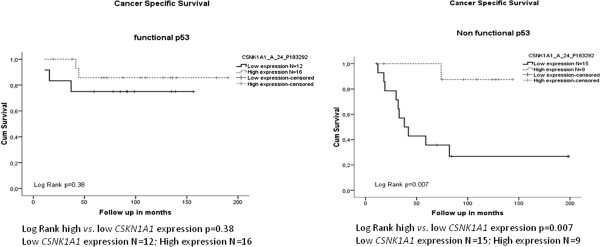
**Kaplan Meier plots for CSS according to *CSNK1A1 *expression stratified on the base of p53 functionality.**

*CSNK1A1* expression significantly altered the effect of p53 in survival as shown in Figure 5. *CSNK1A1* had no influence on survival when p53 is functional, however, when p53 was non-functional, *CSNK1A1* expression influenced disease outcome dramatically. Patients with low *CSNK1A1* expression had a very poor prognosis compared with patients with high *CSNK1A1* expression (Log rank p=0.007) (Figure 5).

**Figure 6:**
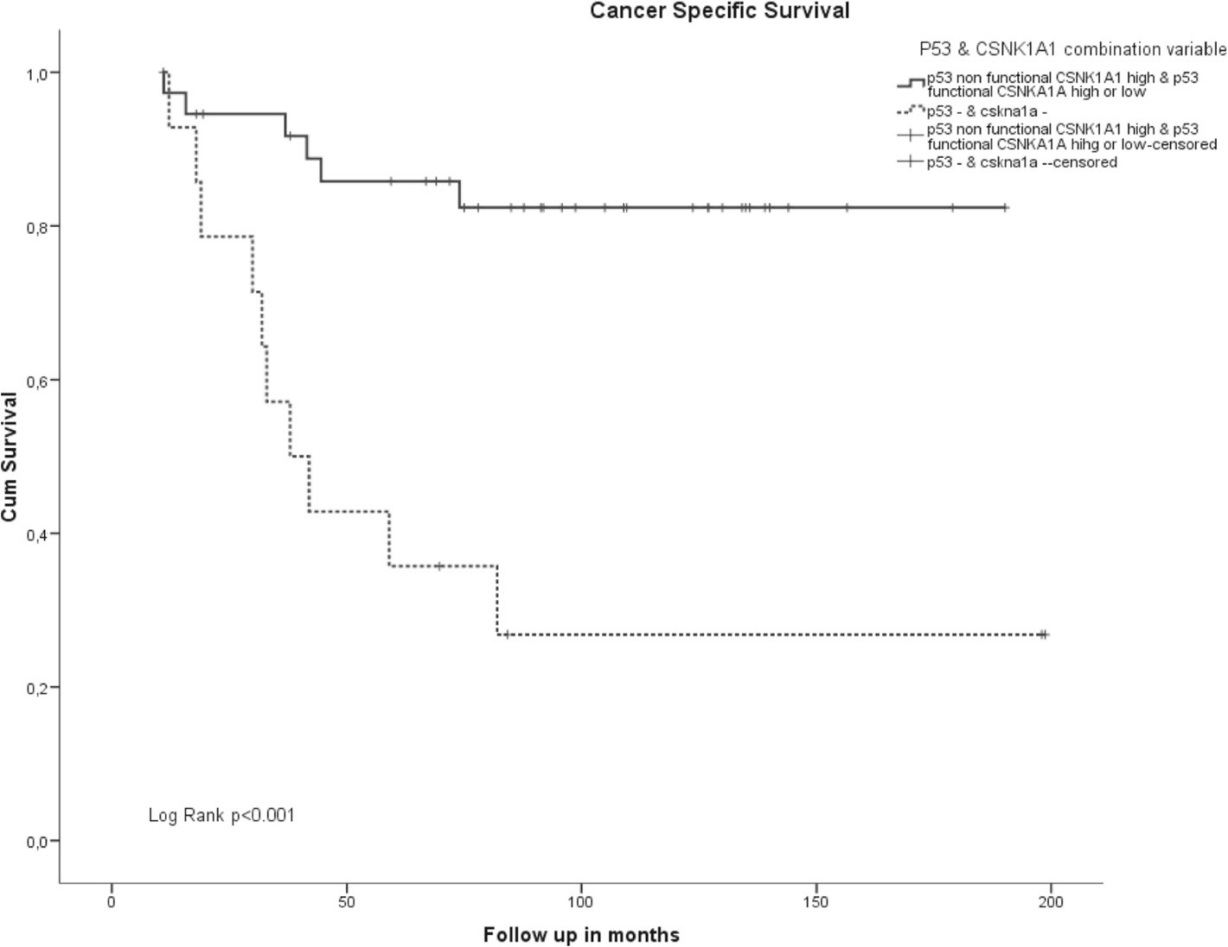
**Kaplan Meier for CSS according to p53 and *CSNK1A1 *combination variable.**

Subsequently, we compared the patients with non functional p53 and low *CSNK1A1* expression with the rest of patients (i.e. non functional p53 and high *CSNK1A1* expression or functional p53 with high or low *CSNK1A1* expression). Survival in patients with both genes affected was decreased compared to patients with one of both genes active (Figure 6) (Log rank p<0.001). Moreover, this detrimental effect on disease outcome was significant in a multivariate model including tumor stage, gender, tumor ocation and MMR status in the model (HR=4.74 95% CI 1.47-15.34 p=0.009) (Table 4).

**Table 4:** Cox Proportional Hazards Model: multivariate survival analysis

**Variables**	**HR**	**95% CI**	**p value**
***p53 & CSNK1A1***			
p53 - & CSNK1A1 + and p53+ & CSNKA1A +/−	Referent		
**p53 - CSNK1A1 -**	**4.74**	**1.47 – 15.34**	0.009*
**Tumor stage**			
I & II	Referent		
**III**	**3.48**	**1.08 – 11.2**	0.037*
**Tumor location**			
Right	Referent		
Left	0.92	0.32 – 2.67	0.58
**Gender**			
Male	0.92	0.32 – 2.97	0.88
Female	Referent		
**MMR state**			
MSS	0.43	0.097 – 1.91	0.27
MSI	Referent		

* Statistically significant results.

### Expression of invasiveness genes

Elyada *et al.* reported up regulation of eight genes in *p53* and *Csnk1a1* double knockout mice and their involvement in murine tumor invasiveness [3]. We analysed their expression in our series. Two genes, mainly *PLAT* (plasminogen activator tissue) and *PNLPRP1* (pancreatic lipase related protein 1) were significantly differently expressed between the two groups of patients; the group with low *CSKN1A1* expression and non-functional p53 vs. the remaining group (with functional p53 and high or low *CSKN1A1* expression and non functional p53 and high *CSNK1A1* expression). *PLAT* was upregulated in the latter group (p=0.009) whereas *PNLPRP1* was higher expressed in the non-functional p53 and low *CSNK1A1* expression (p=0.009).

## Discussion

*TP53* is a transcription factor with important functions in cellular apoptosis, senescence, DNA damage repair, autophagy, aging and glycolysis [34-36]. Therefore, it is a strategic target for inactivation in cancer cells; indeed, somatic mutations are found in approximately 50% of all tumors [4]. However, the consequences of p53 inactivation in disease outcome in colon cancer remain controversial and subject to discussion. These inconclusive result could in part be explained by the combination of differences in the techniques used to assess p53 alterations (IHC or mutation analysis), and the many possible ways of p53 inactivation (deletion and dominant negative, loss or gain of function mutations).

We studied *TP53* using several approaches; first we determined tumor ploidy and *TP53* locus allelic state. Next, we assessed *TP53* mutation state and protein expression by IHC. By integrating these data we could predict p53 functionality. The classification in functional and non-functional p53 was supported by the significant differences in target gene expression between these two groups. Thus, with this approach complete information on the gene was obtained allowing a more reliable classification than solely by mutation analysis or immunohistochemistry.

As it could be expected based on the functions of p53, tumors with a non-functional p53 were highly aneuploid. Moreover, the prognosis for patients with these tumors was worse compared to the group with functional p53.

We have also shown that p53 can indeed behave as a haploinsufficient tumor suppressor gene as demonstrated in mouse models [20]. We accurately assessed the *TP53* genotypes by combining the allelic state at the *TP53* locus using SNP arrays, combined with *TP*53 mutation analyses. In our cohort there were a few cases with LOH at the *TP53* locus but without mutations in exons 5, 6, 7 and 8 and without positive immunostaining. Moreover, the tumors had a near-diploid genome and were associated with a good disease outcome as compared with other patients (Supplementary data figure 1). Our finding supports the observation that p53 +/− mice did develop tumors but show a milder phenotype than p53−/− mice [20].

Recently, in mice *Csnk1a1* or *CKIα* expression has been implicated in colon cancer invasiveness and cell transformation in the gut [3]. CSNK1A1 is a serine/threonine kinase that phosphorylates β-catenin to target it for destruction [37]. In a mouse model, ablation of *Csnk1a1* caused the accumulation of β-catenin in the cytoplasm and nucleus activating many *Wnt* target genes although no tumor formation was observed. Instead, senescence was induced in these cells pointing to a possible role of p53 in tumor inhibition. Indeed, the authors found that inactivation of both *Csnk1a1* and *p53* rendered the cell malignant and rapidly invasive [3]. Likewise, in the present cohort of patients, we have identified *CSNK1A1* as a dramatic modifier of p53 effects on survival. High *CSNK1A1* expression partly counteracts the negative effects of a non functional p53. Accordingly, low *CSNK1A1* expression and non functional p53 was equal to a very poor prognosis with a median survival time of 3 years and a 5-year survival of only 35%, which is extremely poor for early stage disease. Furthermore, this negative effect on survival was independent of disease stage, gender, tumor location and mismatch repair state, as shown in the multivariate analysis.

The exact mechanism behind this poor survival is unknown; Elyada *et al.* showed that expression of certain genes was upregulated in the double knockout mice (*p53*−/− and *Csnk1a1*−/−) as compared with the only *Csnk1a1*−/− mice. Some of these genes were involved in loss of enterocyte polarity, tissue remodeling and cell motility; all functions likely to be involved in tumor invasiveness [3]. In the present cohort of patients only two of the human homologues from the murine gene list proposed were differentially expressed, i.e. *plasminogen activator tissue* (*PLAT*) and *pancreatic lipase related protein 1* (*PNLRP1*) in tumors with impaired p53 function and low expression of *CSNK1A1* versus the remaining tumors. The latter results might reflect differences between mouse and man. Moreover, the human comparison was not identical to the murine comparison by Elyada and co workers. Furthermore in contrast to the murine model, *PLAT* was upregulated in the group with at least one active gene (functional p53 with low or high *CSNK1A1* expression and non functional p53 with high *CSNK1A1* expression) and could therefore be associated with a better survival. In human, the increased expression of the plasminogen activator inhibitor was associated with the occurrence of distant metastasis in colon cancer [38], probably leading to decreased levels of *PLAT* which would corroborate our findings. To our knowledge, the role of *PNLRP1* in tumor invasiveness and progression is so far unknown.

## Conclusion

The combination of several approaches provides additional and accurate information on p53 status showing a detrimental effect on survival when p53 function is impaired. Nevertheless, gene interplay remains very important in tumor biology as it is illustrated by the modifying role of *CSNK1A1* gene expression on the survival effects of *TP53* in colon cancer. Loss of both genes confers an extremely poor prognosis to colon cancer patients.

## Competing interest

The authors have no conflict of interest to disclose.

## Authors’ contributions

All authors have contributed equally in the preparation and execution of this manuscript. AFS: data analysis, writing, allelic state assessment according to LAIR algorithm, FISH, p53 mutation analysis and immunohistochemistry scores. GIF: DNA isolation, cell sorting, manuscript review. WEC: allelic scores, cell sorting, manuscript review. NF dM: clinical follow up of the cohort, DNA isolation, immunohistochemistry, MSI determination, manuscript review. DR: allelic state score, statistical and array analysis, concept and manuscritp review. RvE: DNA isolation, p53 mutation and manuscript review. JO: Concept, LAIR algorithm development, statistics and manuscript review. RT: patient selection, concept and manuscript review. TvW: concept, DNA isolation, allelic state score and mnuscript review. HM: concept, analysis of histomorfology and immunohistochemistry scores and manuscript review. All authors read and approved the final manuscript.

## Pre-publication history

The pre-publication history for this paper can be accessed here:

http://www.biomedcentral.com/1471-2407/13/277/prepub

## Supplementary Material

Additional file 1: Table S1 Call of p53 functionality according to all parameters analyzed. Click here for file

## References

[B1] Cho KR Vogelstein B Genetic alterations in the adenoma–carcinoma sequence Cancer 1992 70 6 Suppl 1727 1731 1516027 10.1002/1097-0142(19920915)70:4+<1727::aid-cncr2820701613>3.0.co;2-p

[B2] Bargonetti J Manfredi JJ Multiple roles of the tumor suppressor p53 Curr Opin Oncol 2002 14 1 86 91 10.1097/00001622-200201000-00015 11790986

[B3] Elyada E Pribluda A Goldstein RE Morgenstern Y Brachya G Cojocaru G Snir-Alkalay I Burstain I Haffner-Krausz R Jung S CKIalpha ablation highlights a critical role for p53 in invasiveness control Nature 2011 470 7334 409 413 10.1038/nature09673 21331045

[B4] Hollstein M Sidransky D Vogelstein B Harris CC p53 mutations in human cancers Science 1991 253 49 53 10.1126/science.1905840 1905840

[B5] Baretton GB Vogt M Muller C Diebold J Schneiderbanger K Schmidt M Lohrs U Prognostic significance of p53 expression, chromosome 17 copy number, and DNA ploidy in non-metastasized colorectal carcinomas (stages IB and II) Scand J Gastroenterol 1996 31 5 481 489 10.3109/00365529609006769 8734346

[B6] Bazan V Migliavacca M Zanna I Tubiolo C Corsale S Calo V Amato A Cammareri P Latteri F Grassi N DNA ploidy and S-phase fraction, but not p53 or NM23-H1 expression, predict outcome in colorectal cancer patients. Result of a 5-year prospective study J Cancer Res Clin Oncol 2002 128 12 650 658 10.1007/s00432-002-0394-6 12474051 PMC12164427

[B7] Bleeker WA Hayes VM Karrenbeld A Hofstra RM Hermans J Buys CC Plukker JT Impact of KRAS and TP53 mutations on survival in patients with left- and right-sided Dukes’ C colon cancer Am J Gastroenterol 2000 95 10 2953 2957 10.1111/j.1572-0241.2000.02327.x 11051374

[B8] Bouzourene H Gervaz P Cerottini JP Benhattar J Chaubert P Saraga E Pampallona S Bosman FT Givel JC p53 and Ki-ras as prognostic factors for Dukes’ stage B colorectal cancer Eur J Cancer 2000 36 8 1008 1015 10.1016/S0959-8049(00)00036-8 10885605

[B9] Chang S-C Lin J-K Yang SH Wang H-S Li AF-Y Chi C-W Relationship between genetic alterations and prognosis in sporadic colorectal cancer Int J Cancer 2006 118 1721 1717 10.1002/ijc.21563 16231316

[B10] Clausen OP Lothe RA Borresen-Dale AL De Angelis P Chen Y Rognum TO Meling GI Association of p53 accumulation with TP53 mutations, loss of heterozygosity at 17p13, and DNA ploidy status in 273 colorectal carcinomas Diagn Mol Pathol 1998 7 4 215 223 10.1097/00019606-199808000-00006 9917132

[B11] Conlin A Smith G Carey FA Wolf CR Steele RJ The prognostic significance of K-ras, p53, and APC mutations in colorectal carcinoma Gut 2005 54 9 1283 1286 10.1136/gut.2005.066514 15843421 PMC1774675

[B12] Elsaleh H Powel B McCaul K Grieu F Grant R Joseph D Iacopetta B p53 alteration and microsatellite instability have predictive value for survival benefit from chemotherapy in stage III colorectal carcinoma Clin Cancer Res 2001 7 1343 1349 11350904

[B13] Goh H-S Chan C-S Khine K Smith DR p53 and the behaviour of colorectal cancer Lancet 1994 344 233 234 10.1016/S0140-6736(94)93000-7 7913159

[B14] Goh H-S Yao J Smith DR p53 point mutation and survival in colorectal cancer patients Cancer Res 1995 55 5217 5221 7585578

[B15] Iacopetta B Russo A Bazan V Dardanoni G Gebbia N Soussi T Kerr D Elsaleh H Soong R Kandioler D Functional categories of TP53 mutation in colorectal cancer: results of an International Collaborative Study Ann Oncol 2006 17 5 842 847 10.1093/annonc/mdl035 16524972

[B16] Munro AJ Lain S Lane DP p53 abnormalities and outcomes in colorectal cancer:a systematic review Br J Cancer 2005 92 434 444 15668707 10.1038/sj.bjc.6602358 PMC2362083

[B17] Russo A Bazan V Agense V Rodolico V Gebbia N Prognostic and predictive factors in colorectal cancer: Kirsten Ras in CRC (RASCAL) and TP53CRC collaborative studies Ann Oncol 2005 16 4 iv44 iv49 15923428 10.1093/annonc/mdi907

[B18] Walther A Houlston R Tomlinson I Association between chromosomal instability and prognosis in colorectal cancer: a meta-analysis Gut 2008 57 7 941 950 10.1136/gut.2007.135004 18364437

[B19] Westra JL Boven LG van der Vlies P Faber H Sikkema B Schaapveld M Dijkhuizen T Hollema H Buys CH Plukker JT A substantial proportion of microsatellite-unstable colon tumors carry TP53 mutations while not showing chromosomal instability Genes Chromosomes Cancer 2005 43 2 194 201 10.1002/gcc.20148 15729700

[B20] Venkatachalam S Shi YP Jones SN Vogel H Bradley A Pinkel D Donehower LA Retention of wild-type p53 in tumors from p53 heterozygous mice: reduction of p53 dosage can promote cancer formation EMBO J 1998 17 16 4657 4667 10.1093/emboj/17.16.4657 9707425 PMC1170795

[B21] Lynch CJ Milner J Loss of one p53 allele results in four-fold reduction of p53 mRNA and protein: a basis for p53 haplo-insufficiency Oncogene 2006 25 24 3463 3470 10.1038/sj.onc.1209387 16449974

[B22] Yoon H Liyanarachchi S Wright FA Davuluri R Lockman JC de la Chapelle A Pellegata NS Gene expression profiling of isogenic cells with different TP53 gene dosage reveals numerous genes that are affected by TP53 dosage and identifies CSPG2 as a direct target of p53 Proc Natl Acad Sci USA 2002 99 24 15632 15637 10.1073/pnas.242597299 12438652 PMC137768

[B23] Corver WE Middeldorp A ter Haar NT Jordanova ES van Puijenbroek M van Eijk R Cornelisse CJ Fleuren GJ Morreau H Oosting J Genome-wide allelic state analysis on flow-sorted tumor fractions provides an accurate measure of chromosomal aberrations Cancer Res 2008 68 24 10333 10340 10.1158/0008-5472.CAN-08-2665 19074902

[B24] Salazar R Roepman P Capella G Moreno V Simon I Dreezen C Lopez-Doriga A Santos C Marijnen C Westerga J Gene Expression Signature to Improve Prognosis Prediction of Stage II and III Colorectal Cancer J Clin Oncol 2011 29 1 17 24 10.1200/JCO.2010.30.1077 21098318

[B25] Bosman F Carneiro F Hruban RH Theise ND WHO classification of tumours of the digestive system 2010 Fourth Lyon, France: International Agency for Research on Cancer (IARC) Chapter 8; 134-146

[B26] Corver WE ter Haar NT High-resolution multiparameter DNA flow cytometry for the detection and sorting of tumor and stromal subpopulations from paraffin-embedded tissues Curr Protoc Cytom 2011 7 7 37 21207360 10.1002/0471142956.cy0737s55

[B27] Corver WE Ter Haar NT Dreef EJ Miranda NF Prins FA Jordanova ES Cornelisse CJ Fleuren GJ High-resolution multi-parameter DNA flow cytometry enables detection of tumour and stromal cell subpopulations in paraffin-embedded tissues J Pathol 2005 206 2 233 241 10.1002/path.1765 15822070

[B28] Lips EH van Eijk R de Graaf EJ Doornebosch PG de Miranda NF Oosting J Karsten T Eilers PH Tollenaar RA van Wezel T Progression and tumor heterogeneity analysis in early rectal cancer Clin Cancer Res 2008 14 3 772 781 10.1158/1078-0432.CCR-07-2052 18245538

[B29] Middeldorp A van Eijk R Oosting J Forte GI van Puijenbroek M van Nieuwenhuizen M Corver WE Ruano D Caldes T Wijnen J Increased frequency of 20q gain and copy-neutral loss of heterozygosity in mismatch repair proficient familial colorectal carcinomas Int J Cancer 2011 10.1002/ijc.26093 21445971

[B30] Cruz I Snijders PJ Van Houten V Vosjan M Van der Waal I Meijer CJ Specific p53 immunostaining patterns are associated with smoking habits in patients with oral squamous cell carcinomas J Clin Pathol 2002 55 11 834 840 10.1136/jcp.55.11.834 12401821 PMC1769794

[B31] Romeo S Debiec-Rychter M Van Glabbeke M Van Paassen H Comite P Van Eijk R Oosting J Verweij J Terrier P Schneider U Cell cycle/apoptosis molecule expression correlates with imatinib response in patients with advanced gastrointestinal stromal tumors Clin Cancer Res 2009 15 12 4191 4198 10.1158/1078-0432.CCR-08-3297 19509155

[B32] Hosmer DW Lemeshow S Applied survival analysis. Regression modelling of time to event data 1999 New York: John Wiley & Sons, INC

[B33] Smyth GK Linear models and empirical bayes methods for assessing differential expression in microarray experiments Stat Appl Genet Mol Biol 2004 3 3 10.2202/1544-6115.1027 16646809

[B34] Vousden KH Lane DP p53 in health and disease Nat Rev Mol Cell Biol 2007 8 4 275 283 10.1038/nrm2147 17380161

[B35] Vousden KH Prives C Blinded by the Light: The Growing Complexity of p53 Cell 2009 137 3 413 431 10.1016/j.cell.2009.04.037 19410540

[B36] Zuckerman V Wolyniec K Sionov RV Haupt S Haupt Y Tumour suppression by p53: the importance of apoptosis and cellular senescence J Pathol 2009 219 1 3 15 19562738 10.1002/path.2584

[B37] Liu C Li Y Semenov M Han C Baeg GH Tan Y Zhang Z Lin X He X Control of beta-catenin phosphorylation/degradation by a dual-kinase mechanism Cell 2002 108 6 837 847 10.1016/S0092-8674(02)00685-2 11955436

[B38] Markl B Renk I Oruzio DV Jahnig H Schenkirsch G Scholer C Ehret W Arnholdt HM Anthuber M Spatz H Tumour budding, uPA and PAI-1 are associated with aggressive behaviour in colon cancer J Surg Oncol 2010 102 3 235 241 10.1002/jso.21611 20740581

