# Supplementary material for: Integral analysis of p53 and its value as prognostic factor in sporadic colon cancer
Source: BMC Cancer. 2013 Jun 5;13:277. doi: 10.1186/1471-2407-13-277 (PMC3682902; doi:10.1186/1471-2407-13-277)
Supplement: Additional file 1: Table S1 — Call of p53 functionality according to all parameters analyzed. [file 1471-2407-13-277-S1.doc]

| **Sample** | ***TP53* mutation** | **p53 IHC** | **Chr.17 allelic status** | **p53 functionality** |
| --- | --- | --- | --- | --- |
| 1 | mut | >25% | AAAA | impaired |
| 2 | mut | 0 | AAB | impaired |
| 3 | wt | >25% | AB | impaired |
| 4 | mut | >25% | AA | impaired |
| 5 | wt | <25% | AB | unimpaired |
| 6 | unknown | <25% | AB | unimpaired |
| 7 | unknown | <25% | AB | unimpaired |
| 8 | wt | <25% | AB | unimpaired |
| 9 | wt | <25% | AB | unimpaired |
| 10 | unknown | >25% | AB + AAAA | impaired |
| 11 | unknown | <25% | AB | unimpaired |
| 12 | wt | <25% | AB + AA | unimpaired |
| 13 | unknown | 0 | AB | unimpaired |
| 14 | unknown | >25% | AB + AA | impaired |
| 15 | wt | <25% | AB | unimpaired |
| 16 | unknown | <25% | AB | unimpaired |
| 17 | wt | 0 | A | unimpaired |
| 18 | unknown | 0 | A | unimpaired |
| 19 | wt | <25% | AB | unimpaired |
| 20 | wt | 0 | A | unimpaired |
| 21 | mut | >25% | AA | impaired |
| 22 | mut | >25% | AA + AB | impaired |
| 23 | wt | <25% | AB | unimpaired |
| 24 | mut | >25% | AA | impaired |
| 25 | wt | 0 | AB | unimpaired |
| 26 | unknown | >25% | A | impaired |
| 27 | mut | 0 | AA | impaired |
| 28 | wt | <25% | A | unimpaired |
| 29 | mut | <25% | AB | impaired |
| 30 | mut (non functional mutation) | 0 | AB +AA | unimpaired |
| 31 | unknown | <25% | AB | unimpaired |
| 32 | unknown | <25% | AB | unimpaired |
| 33 | mut (non functional mutation) | <25% | AB | unimpaired |
| 34 | mut (non functional mutation) | <25% | AB | unimpaired |
| 35 | unknown | >25% | AA | impaired |
| 36 | wt | >25% | AB + AB | impaired |
| 37 | unknown | <25% | AB | unimpaired |
| 38 | unknown | >25% | AB | impaired |
| 39 | unknown | >25% | AAAAA | impaired |
| 40 | mut | >25% | AB | impaired |
| 41 | mut | >25% | AA | impaired |
| 42 | mut | >25% | AB + AA | impaired |
| 43 | wt | >25% | AAA | impaired |
| 44 | mut (non functional mutation) | <25% | AB | unimpaired |
| 45 | wt | <25% | AB | unimpaired |
| 46 | mut | >25% | AA | impaired |
| 47 | wt | >25% | AB + AA | impaired |
| 48 | wt | >25% | AAA | impaired |
| 49 | unknown | <25% | AB | unimpaired |
| 50 | unknown | >25% | AAAA | impaired |
| 51 | mut | >25% | AB | impaired |
| 52 | wt | 0 | AA | impaired |
| 53 | wt | <25% | AB | unimpaired |
| 54 | wt | >25% | A | impaired |
| 55 | mut | >25% | AA | impaired |
| 56 | wt | 0 | AAA | unimpaired |
| 57 | unknown | <25% | AB | unimpaired |

Table: p53 integrity for each sample
